# Plasma VP8∗-Binding Antibodies in Rotavirus Infection and Oral Vaccination in Young Bangladeshi Children

**DOI:** 10.1093/jpids/piab120

**Published:** 2021-12-14

**Authors:** Benjamin Lee, E Ross Colgate, Marya Carmolli, Dorothy M Dickson, Soyeon Gullickson, Sean A Diehl, Rifat Ara, Masud Alam, Golam Kibria, Md Abdul Kader, Sajia Afreen, Tahsin Ferdous, Rashidul Haque, Beth D Kirkpatrick

**Affiliations:** 1 Department of Pediatrics, Division of Pediatric Infectious Diseases, University of Vermont Larner College of Medicine, Burlington, Vermont, USA; 2 Translational Global Infectious Diseases Research Center, University of Vermont Larner College of Medicine, Burlington, Vermont, USA; 3 Department of Microbiology and Molecular Genetics, University of Vermont Larner College of Medicine, Burlington, Vermont, USA; 4 Department of Parasitology and Emerging Infections, International Centre for Diarrhoeal Disease Research, Dhaka, Bangladesh

**Keywords:** correlates of protection, oral vaccines, rotavirus, rotavirus vaccines, viral gastroenteritis

## Abstract

**Background:**

Despite the availability and success of live-attenuated oral vaccines, rotavirus (RV) remains the leading cause of pediatric gastroenteritis worldwide. Next-generation vaccines targeting RV VP8∗ are under evaluation, but the role of VP8∗-specific antibodies in human immunity to RV and their potential as immune correlates of protection remains underexplored.

**Methods:**

We measured plasma RV VP8∗-binding antibodies in 2 cohorts of young children in Dhaka, Bangladesh. Plasma from a cohort study of 137 unvaccinated children aged 6-24 months old hospitalized with acute gastroenteritis was assessed for VP8∗ antibody seropositivity. VP8∗ antibodies were compared with the current standard for RV immunity, total RV-specific IgA (RV-IgA). Additionally, VP8∗ antibody responses were measured as part of an immunogenicity trial of a monovalent, oral, live-attenuated RV vaccine (Rotarix).

**Results:**

Fewer children with acute RV gastroenteritis were seropositive for VP8∗-binding IgA or IgG antibodies at hospital admission compared with RV-IgA, suggesting that the absence of VP8∗-binding antibodies more accurately predicts susceptibility to RV gastroenteritis than RV-IgA in unvaccinated children. However, when present, these antibodies appeared insufficient to protect fully from disease and no threshold antibody level for protection was apparent. In vaccinated children, these antibodies were very poorly induced by Rotarix vaccine, suggesting that VP8∗-specific antibodies alone are not necessary for clinical protection following oral vaccination.

**Conclusions:**

This work suggests that VP8∗-binding antibodies may not be sufficient or necessary for protection from RV gastroenteritis following prior RV infection or oral vaccination; the role of VP8∗ antibodies induced by parenteral vaccination with non-replicating vaccines remains to be determined.

## Teaser

Plasma antibodies binding the rotavirus VP8∗ structural protein were insufficient to fully protect from rotavirus gastroenteritis and were poorly induced by the oral, live-attenuated vaccine Rotarix in young children in Dhaka, Bangladesh.

Rotavirus (RV) remains the leading cause of diarrheal disease and mortality in children, despite the availability and significant successes of live-attenuated, oral RV vaccines (ORVs) [1, 2]. To further decrease the impact of childhood diarrhea in low- and middle-income countries (LMICs) where disease burden is highest, strategies to improve existing vaccine performance and development of next-generation vaccines are required. Such efforts would be aided by greater understanding of the mechanisms conferring protection from diarrhea and identification of improved immune correlates of protection (CoP). Total serum RV-specific Immunoglobulin A (RV-IgA) is the current standard for measuring RV immunity [3]. However, this measure is a suboptimal CoP in LMICs [4].

RVs are traditionally classified by the 2 outer capsid structural proteins, VP7 (G, glycoprotein) and VP4 (P, protease-sensitive spike), both of which elicit neutralizing antibodies [5]. The middle capsid layer consists of VP6, which is immunodominant but does not elicit neutralizing antibodies. VP6 is the main target in the RV-IgA assay, but because it is chiefly recognized during viral replication, it may not be a relevant immune marker for non-replicating parenteral vaccines. VP4 is composed of 2 subunits, VP8∗ and VP5∗, which are exposed following proteolysis by intestinal trypsin [6]. VP8∗ determines P-type specificity; binds to cellular receptors and host-derived antigens, including histoblood group antigens; induces protective neutralizing antibodies in animal models; and is easily expressed in cell culture [7-10].

For these reasons, there is considerable interest in development of VP8∗ antigen-based, non-replicating parenteral vaccines [11]. However, surprisingly little is known regarding the contribution of VP8∗-binding antibodies to protection from RV gastroenteritis in children, or their induction by currently approved ORVs. Therefore, we explored plasma VP8∗-specific antibodies in young children in Dhaka, Bangladesh, with acute RV gastroenteritis or following oral vaccination with a monovalent (G1P[8]) ORV (Rotarix, GlaxoSmithKline).

## METHODS

### Study Design

#### Natural Infection Study

We conducted a cohort study among children aged 6-24 months admitted for acute gastroenteritis to the International Centre for Diarrhoeal Disease Research, Bangladesh (icddr,b), in Dhaka between February and July 2018. Inclusion criteria were age 6-24 months, hospitalization for acute gastroenteritis (defined as ≥3 abnormally loose bowel movements or ≥1 episode of forceful vomiting per 24-hour period with duration of ≤7 days at the time of admission), and willingness to adhere to study procedures. Exclusion criteria included chronic illness, severe malnutrition, known immunocompromising condition, prior RV vaccination, or other household member enrolled in the study. RV vaccine was not widely available at the time of the study. The study was approved by the Ethical/Institutional Review Boards of the icddr,b and the University of Vermont. All participating families provided written informed consent.

Blood and stool were collected at enrollment (day 0) and in follow-up at the study clinic or via home visit (days 7, 28). The primary outcome was day 0 plasma IgA targeting Rotarix vaccine-strain VP8∗ (VP8∗-IgA), and the primary objective was comparison of the frequency of seropositivity (as defined below) for day 0 VP8∗-IgA and RV-IgA in children with RV gastroenteritis, diagnosed by stool RV detection using real-time reverse transcription (RT) quantitative polymerase chain reaction (qPCR) in day 0 stool. We also conducted a nested, test-negative case-control study, with cases defined as RV enzyme immunoassay (EIA)+ children and controls as RV EIA− children. Secondary outcomes included VP8∗-IgG measurement, assessment based on, and comparison of antibodies between children with and without RV gastroenteritis.

The study was powered to detect a 20% difference in the proportion of children with RV gastroenteritis who were seropositive for RV-IgA vs VP8∗-IgA at 80% power and alpha = 0.05. Based on prior unpublished data, we assumed 50% of admitted children would have RV gastroenteritis, 25% of whom would be RV-IgA seropositive at admission. We hypothesized that the presence of VP8∗ antibodies would be a marker of a more protective immune response to prior RV infection than RV-IgA and thus assumed that fewer children admitted for RV gastroenteritis would be seropositive for VP8∗-IgA than RV-IgA (5% vs 25%). We estimated that this would require a minimum of 78 RV-infected children. To monitor accrual in real time, we initially identified RV-infected children using RV antigen EIA (Oxoid Ltd., Hampshire, England), with the study designed to halt enrollment once 78 RV EIA+ cases were enrolled.

Basic information regarding demographics, socioeconomic status, and symptoms were collected via structured questionnaire at enrollment and follow-up. Study data were managed using REDCap electronic data capture tools hosted at the University of Vermont [12, 13].

#### Rotarix Immunogenicity Study

We measured post-Rotarix (G1[P8]) VP8∗-IgA and VP8∗-IgG in a subset of infants who participated in a randomized, controlled immunogenicity trial of infants randomized to receive either a single standard dose or 2 simultaneous doses (twice-standard dose) Rotarix at 6 and 10 weeks of life. Full study results have been published [14]. Blood was collected at 6 weeks (pre-vaccination) and 14 weeks (post-vaccination). Vaccine inoculum had no impact on overall vaccine take; therefore, infants were analyzed here irrespective of study arm.

### Rotavirus Detection and P-Typing

In the Natural Infection Study, stool RV EIA was performed on day 0 stool immediately following enrollment. Subsequently, total nucleic acid extraction followed by RV RNA detection by qPCR on all stool specimens with P-type identification of positive specimens via Sanger sequencing was performed as previously described [14]. Sequences were analyzed using Geneious Prime, version 2019.2.1 (Biomatters Ltd., Auckland, New Zealand).

### VP8∗ Antibody Measurement

To detect RV VP8∗-IgA and -IgG antibodies in human plasma, we developed an indirect enzyme-linked immunosorbent assay (ELISA) using recombinant Rotarix vaccine-strain P[8] VP8∗ as antigen. Detailed methods are provided in the Supplementary Methods. Plasma VP8∗-IgA and VP8∗-IgG were measured at days 0 and 28 in all Natural Infection Study participants. Seropositivity was defined as detection of any VP8∗-IgA or VP8∗-IgG. For the Rotarix immunogenicity study, we evaluated participants with RV-IgA seroconversion, defined as seronegative pre-vaccination (concentration <20 U/mL) and seropositive post-vaccination (concentration ≥20 U/mL), as previously described [4], or with demonstrated rise in RV-IgG concentration, measured by ELISA (Supplementary Methods).

Because the effect of VP8∗-binding antibodies on infection might be P-type specific, we screened for P[4]- and P[6]-VP8∗-binding antibodies in participants with sequence-confirmed P[4] or P[6] RV infections with an end-point assay using purified, recombinant antigens consisting of truncated strain DS-1 P[4] VP8∗ or strain 1076 P[6] VP8∗ fused to the tetanus toxin epitope P2 [15]. These were a kind gift from Stan Cryz and Alan Fix (PATH). Detailed methods are provided in the Supplementary Methods.

### Statistical Analysis

The proportion of cases seropositive for RV-IgA vs VP8∗-IgA at day 0 was assessed using McNemar’s test. Comparison of categorical outcomes was analyzed by Chi-square or Fisher’s exact test, as appropriate, with calculation of odds ratios (OR) and associated 95% confidence intervals (CI). Continuous variables were compared by Mann-Whitney *U*-test or independent samples *t*-test as appropriate based on data distribution. Antibody concentrations were log-transformed for analysis. Correlations between continuous variables were assessed using linear regression or Spearman’s rank correlation, as appropriate based on data distribution. All analyses were performed using SPSS version 27 (IBM Corps., Armonk, NY) or GraphPad Prism version 8.2.1 (GraphPad Software, San Diego, CA).

## RESULTS

In the Natural Infection Study, 78 RV EIA+ and 59 RV EIA− children were enrolled (N = 137), of whom 73 EIA+ and 57 EIA− children had evaluable data. Ten EIA− children were qPCR+ at day 0, and all EIA+ children were qPCR+. The highest cycle threshold (Ct) for day 0 qPCR detections was 30.8, lower than the cutoff of 32.6 previously demonstrated to be highly associated with RV gastroenteritis [16], suggesting that these infections were true cases of RV gastroenteritis and not asymptomatic RV coinfection with an alternate cause for diarrhea. Therefore, results are presented with RV gastroenteritis defined as day 0 qPCR+ (N = 83) vs qPCR− (N = 47).

Select baseline characteristics are presented in **Table 1**. No significant differences were detected between children with or without RV gastroenteritis, except RV+ children were younger (*P* = .024) and less likely to live near an open drain (*P* = .014). No age difference was noted when RV gastroenteritis was defined by EIA detection, meaning the additional cases identified by qPCR were younger, reflecting the well-established observation that RV incidence is highest in younger children in unvaccinated populations. Most RV cases had P[8] infection (N = 62), followed by P[4] (N = 13) and P[6] (N = 5); typing was unsuccessful in 3 samples.

**Table 1.** Selected Baseline Characteristics of Children Admitted to icddr,b Hospital for Acute Gastroenteritis

Enrollment Characteristics^a^	RV qPCR Positive N = 83	RV qPCR Negative N = 47	Total N = 130
Age in months, mean (SD)^b^	11.0 (4.2)	12.9 (5.2)	11.7 (4.6)
Sex, female	33 (40%)	17 (36%)	50 (39%)
Weight-for-age *Z* score, mean (SD)	−0.92 (0.13)	−1.16 (0.18)	−1.00 (0.11)
Height-for-age *Z* score, mean (SD)	−0.61 (0.14)	−1.02 (0.21)	−0.76 (0.12)
Vaginal birth	40 (48%)	21 (45%)	61 (47%)
Home birth	22 (27%)	8 (17%)	30 (23%)
First child	37 (45%)	21 (45%)	58 (45%)
Open drain near home^c^	26 (31%)	25 (53%)	51 (39%)
Piped water source	68 (82%)	38 (81%)	106 (82%)
No form of water treatment	22 (27%)	12 (26%)	34 (26%)
Monthly household income (Taka),^d^ median (IQR)	20 000 (15 000-25 000)	20 000 (15 000-25 000)	20 000 (15 000-25 000)
Days of diarrhea at admission, median (IQR)	3 (2-5)	3 (2-4)	3 (2-4)

Abbreviations: icddr,b, International Centre for Diarrhoeal Disease Research, Bangladesh; IQR, interquartile range; RV, rotavirus; qPCR, quantitative polymerase chain reaction; SD, standard deviation.

Data presented as N (%) except as otherwise noted.

*P =* .024.

*P* = .014.

At the time of study, ~84 Taka = 1 USD.

### Day 0 VP8∗ vs RV-IgA Antibodies in Children With RV Gastroenteritis

Among children with RV gastroenteritis, 8% (N = 7/83) were seropositive for VP8∗-IgA at day 0, compared with 52% (N = 43/83) for RV-IgA (*P* = 2.8 × 10^−10^; **Table 2**). Similarly, 11% (N = 9) of children with RV gastroenteritis were VP8∗-IgG seropositive at day 0, compared with 52% for RV-IgA (*P* = 1.1 × 10^−9^; **Table 2**). Among the 7 children seropositive for VP8∗-IgA, 6 were also seropositive for VP8∗-IgG and 1 was seronegative. Three children were VP8∗-IgG seropositive but VP8∗-IgA seronegative. Neither VP8∗-IgA nor VP8∗-IgG concentration at day 0 was associated with the number of days after onset of diarrhea (DOD) at the time of specimen collection (Spearman’s rho = −0.050 and −0.113, respectively), suggesting that day 0 antibodies reflected baseline (ie, pre-hospitalization) antibody status, not rapid antibody induction. RV-IgA concentration was positively correlated with DOD (Spearman’s rho, 0.409; *P* = .00012), suggesting more rapid induction of RV-IgA following RV infection than VP8∗-IgA or –VP8∗IgG. When limiting to children with DOD ≤ 3 days at specimen collection (N = 45), this correlation was no longer detected (Spearman’s rho, 0.163; *P* = .29). About 11% (N = 5) were seropositive for VP8∗-IgA at day 0, compared with 36% (N = 16) for RV-IgA (*P* = .001), and 16% (N = 7) were seropositive for VP8∗-IgG at day 0, compared with 36% (N = 16) for RV-IgA (*P* = .012). Among the 7 RV+ children who were VP8∗-IgA seropositive at day 0, 3 had P[8] infections and 4 had P[4] infections, one of whom also was seropositive for P[4]-VP8∗-IgA (end-point titer ≥1:640).

**Table 2.** VP8∗ vs RV-IgA Antibodies at Enrollment in Children Hospitalized for RV Gastroenteritis

	RV-IgA seropositive	RV-IgA seronegative	Total	*P*-value
VP8∗-IgA seropositive	6	1	7 (8%)	2.8 × 10^-10^
VP8∗-IgA seronegative	37	39	76 (92%)	
Total	43 (52%)	40 (48%)	83 (100%)	
VP8∗-IgG seropositive	8	1	9 (11%)	1.1 × 10^-9^
VP8∗-IgG seronegative	35	39	74 (89%)	
Total	43 (52%)	40 (48%)	83 (100%)	

Abbreviation: RV, rotavirus.

Consistent with our hypothesis, these data indicate that the absence of preexisting plasma VP8∗-binding antibodies was more highly associated with susceptibility to RV gastroenteritis than RV-IgA in unvaccinated children. However, preexisting plasma VP8∗-IgA and VP8∗-IgG antibodies were not sufficient to protect children from severe RV gastroenteritis, although data should be interpreted with caution since sample sizes were small.

### VP8∗ Antibody Kinetics in Natural RV Infection

**Figure 1.**
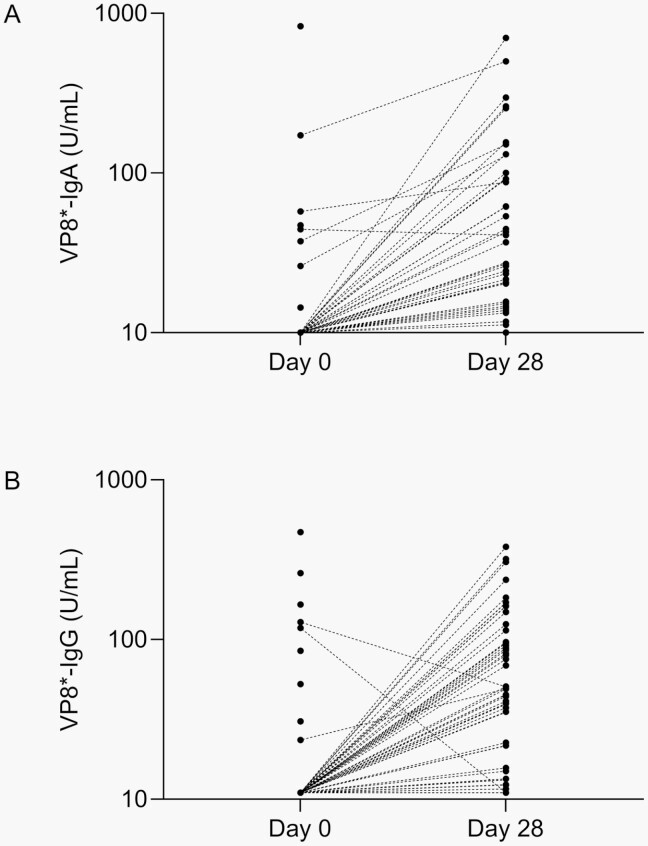
Kinetics of VP8∗-IgA and VP8∗-IgG induction in children with RV infection. Concentrations are given in arbitrary units (U/mL). Because different reference standards were required for each assay, absolute concentrations are not directly comparable between assays. (A) VP8∗-IgA was measured at days 0 and 28 in children with qPCR+ RV infection at either day 0, 7, or 28; N = 92 at day 0, N = 74 at day 28. (B) VP8∗-IgG was measured at days 0 and 28 in children with qPCR+ RV infection at either day 0, 7, or 28; N = 92 at day 0, N = 73 at day 28. Abbreviations: RV, rotavirus; qPCR, quantitative polymerase chain reaction.

We then evaluated the kinetics of VP8∗-IgA and VP8∗-IgG∗ in children with qPCR+ RV infection at any time point (day 0, 7, or 28). Virtually all children with qPCR+ RV infection during the study period demonstrated an increase in VP8∗-IgA (**Figure 1A**) and VP8∗-IgG concentration (**Figure 1B**) from day 0 to 28, demonstrating that this assay detected VP8∗ antibodies at physiologically relevant concentrations and that low day 0 seropositivity rates were unlikely to solely be due to limitations in assay sensitivity. The degree of VP8∗-IgA and VP8∗-IgG antibody induction (measured as fold-rise in concentration from day 0 to 28) was not correlated with duration of hospitalization (data not shown). We conclude that VP8∗-IgA and VP8∗-IgG are reliably induced by RV gastroenteritis and serve as reasonably sensitive markers for recent RV infection.

### VP8∗ Antibodies in Children Hospitalized for RV vs Non-RV Gastroenteritis

**Figure 2.**
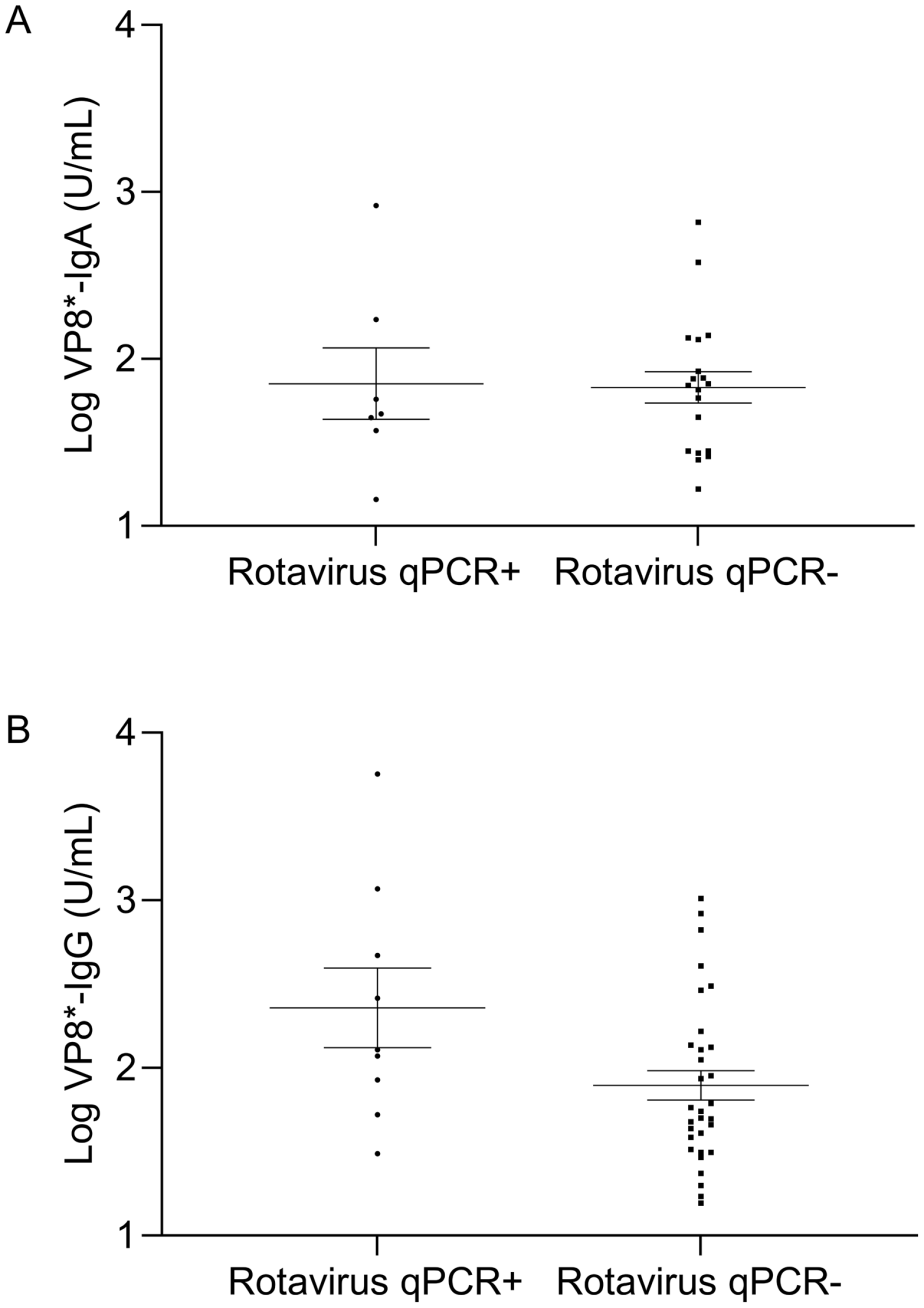
Preexisting VP8∗-IgA and VP8∗-IgG in children hospitalized with RV+ and RV− acute gastroenteritis. Lines and error bars represent GMC with 95% CI. Concentrations are given in arbitrary units (U/mL). Because different reference standards were required for each assay, absolute concentrations are not directly comparable between assays. (A) VP8∗-IgA concentration at day 0 in seropositive children with acute RV gastroenteritis (N = 7, GMC 71.2 U/mL, 95% CI 21.3-238.0) or non-RV gastroenteritis (N = 19, GMC 67.6 U/mL, 95% CI 42.9-106.6; *P* = .9). (B) VP8∗-IgG concentration at day 0 in seropositive children with acute RV gastroenteritis (N = 9, GMC 228.6 U/mL, 95% CI 64.9-805.1) or non-RV gastroenteritis (N = 31, GMC 78.7 U/mL, 95% CI 51.9-119.5; *P* = .03). Abbreviations: RV, rotavirus; GMC, geometric mean antibody concentration; CI, confidence intervals.

Compared with children admitted for non-RV gastroenteritis, significantly fewer RV+ children were seropositive at day 0 for VP8∗-IgA (OR 0.136, 95% CI 0.052-0.358) or VP8∗-IgG (OR 0.063, 95% CI 0.025-0.157; **Table 3**), indicating that the absence of VP8∗ antibodies was strongly associated with hospitalization for RV gastroenteritis in unvaccinated children. However, among all children seropositive for VP8∗-IgA antibodies at day 0, the geometric mean antibody concentration (GMC) was similar in RV+ children (71.2 U/mL, 95% CI 21.3-238.0) compared with RV− children (67.6 U/mL, 95% CI 42.9-106.6; **Figure 2A**). Among all children seropositive for VP8∗-IgG, GMC was actually significantly higher (*P* = .03) in RV+ children (228.6 U/mL, 95% CI 64.9-805.1) compared with RV− children (78.4 U/mL, 95% CI 51.9-119.5; **Figure 2B**).

**Table 3.** VP8∗-Antibody Seropositivity at Enrollment in Children hospitalized for RV and Non-RV Gastroenteritis

	RV qPCR positive N = 83	RV qPCR negative N = 47	OR (95% CI)
VP8∗-IgA seropositive	7 (8%)	19 (40%)	0.136 (0.052-0.358)
VP8∗-IgA seronegative	76 (92%)	28 (60%)	
VP8∗-IgG seropositive	9 (11%)	31 (66%)	0.063 (0.025-0.157)
VP8∗-IgG seronegative	74 (89%)	16 (34%)	

Abbreviations: RV, rotavirus; qPCR, quantitative polymerase chain reaction; OR, odds ratios; CI, confidence intervals.

### VP8∗ Antibodies Following Oral Rotarix Vaccination in Bangladeshi Infants

Next, we assessed the induction of VP8∗-IgA and VP8∗-IgG in Bangladeshi infants in the Rotarix (G1P[8]) immunogenicity study. We assumed that post-vaccination VP8∗-IgA would be most easily detected in infants with total RV-IgA seroconversion following vaccination (N = 82, 44%), as this was previously shown to be associated with viral replication [14] which would presumably increase the overall exposure of VP8∗ to the humoral compartment. Oral Rotarix vaccination almost completely failed to induce VP8∗-IgA in this population: only 3 participants (4%) with RV-IgA seroconversion were seropositive for VP8∗-IgA at week 14, with measured VP8∗-IgA concentrations of 12.3, 77.3, and 105.0 U/mL.

**Figure 3.**
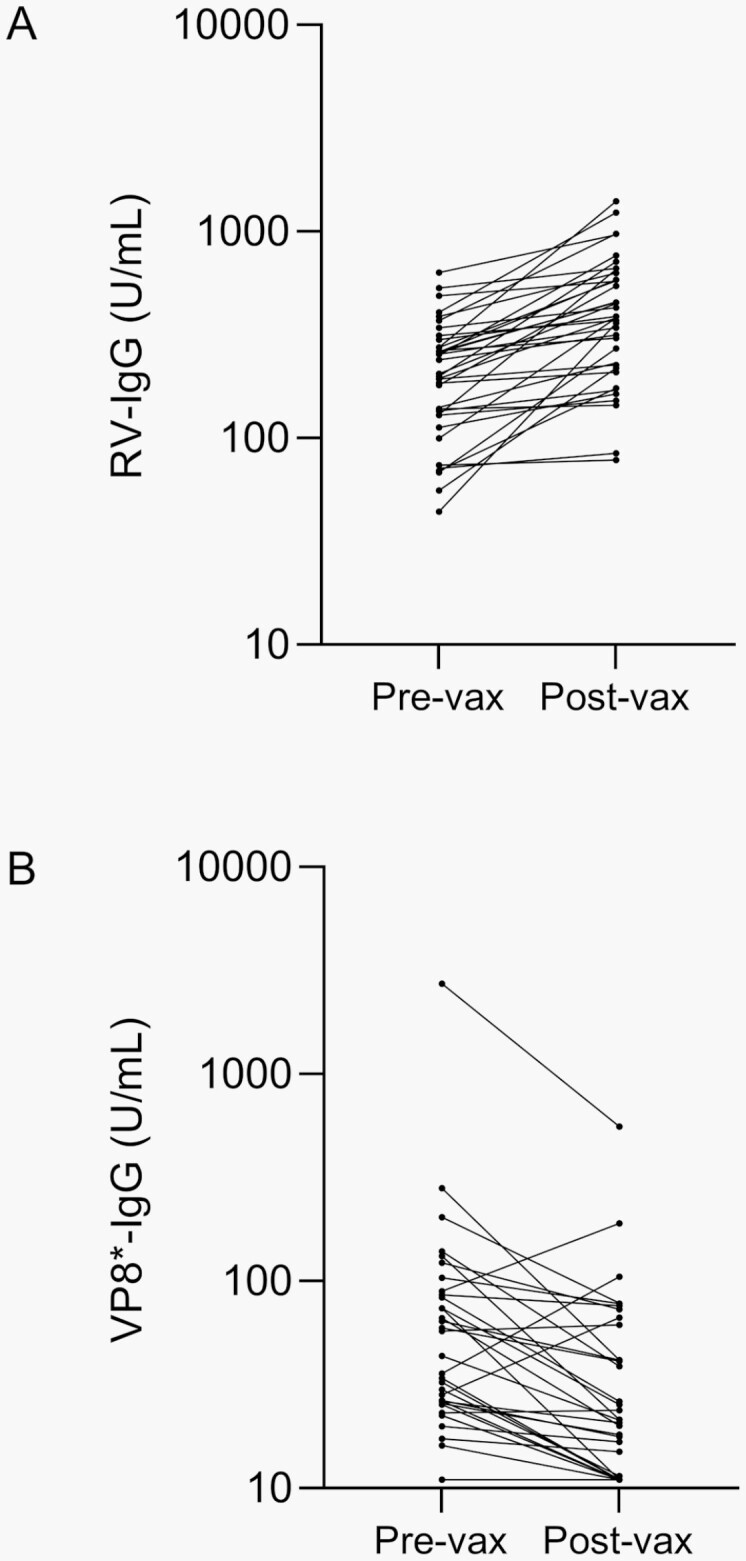
Kinetics of total RV-IgG and VP8∗-IgG antibodies in children pre and post Rotarix vaccination. Concentrations are given in arbitrary units per mL (U/mL). Because different reference standards were required for each assay, absolute concentrations are not directly comparable between assays. (A) In the Rotarix immunogenicity study, N = 37 vaccinated infants (17%) demonstrated rise in total RV-IgG concentration between week 6 (pre-vax) and week 14 (post-vax) of life. (B) Among RV-IgG responders, N = 5 (14%) demonstrated concurrent rise in VP8∗-IgG. Abbreviation: RV, rotavirus.

Since assessment of IgG in young infants is confounded by maternally derived, transplacentally acquired antibodies, for VP8∗-IgG measurement, we selected participants who demonstrated a rise in total RV-IgG between weeks 6 and 14 (N = 37, 17%; **Figure 3A**). This strategy would identify infants exhibiting robust IgG responses to Rotarix, although would miss those with milder responses that were insufficient to be detected amid waning maternal antibodies. Only 5 (14%) infants with rise in total RV-IgG demonstrated concurrent rise in VP8∗-IgG (**Figure 3B**). These data suggest that the contribution of VP8∗-IgG to the overall IgG response to Rotarix in this setting is minimal.

## DISCUSSION

In Bangladesh, very few children admitted for severe RV gastroenteritis were seropositive for VP8∗ antibodies early during acute infection, while over half were seropositive for the current standard marker for RV immunity, total RV-IgA. This suggests that prior infection did not generate immunity against subsequent RV gastroenteritis. The absence of preexisting plasma VP8∗ antibodies was more highly associated with susceptibility to severe RV infection than RV-IgA. And while the presence of these antibodies was associated with decreased odds of RV gastroenteritis, they were insufficient to fully protect against disease. Adapting the nomenclature proposed by Plotkin and Gilbert for vaccine-induced immunity to the natural infection context [17, 18], these antibodies may function similarly to RV-IgA as non-mechanistic, relative CoPs for RV [19]. Because children remained susceptible to RV gastroenteritis at similar antibody concentrations as RV− controls, VP8∗ antibodies alone do not appear to be the mechanistic effector that confers protection. Similarly, there was no threshold concentration clearly associated with protection, similar to RV-IgA [4, 20]. Thus VP8∗-binding antibodies may represent a marker for other effector mechanisms that remain undefined. Finally, these antibodies were very poorly induced by oral Rotarix vaccine (even at double the standard dose), strongly suggesting that the generation of immunity to RV gastroenteritis following successful oral vaccination with live-attenuated (ie, replicating) vaccines in this setting is independent of VP8∗-binding antibodies.

While natural RV infection consistently induced VP8∗-binding antibodies, we were surprised at how poorly these antibodies were induced by Rotarix in Bangladeshi infants. Only 4% of infants with proven total RV-IgA seroconversion (44% of all vaccinated infants in the Rotarix immunogenicity study) [14] were seropositive for VP8∗-IgA following vaccination. Given such low rates of induction in this population, VP8∗-binding antibodies appear to offer no advantage in LMICs as a CoP for ORV trials over the current standard, total RV-IgA. While our Rotarix immunogenicity study did not evaluate vaccine efficacy, previous work in Bangladesh has demonstrated Rotarix 1-year efficacy against RV gastroenteritis of any severity of 51% in a delayed-dosing randomized clinical trial [21], and 1-year effectiveness of 45.2% in a cluster-randomized trial using typical dosing schedules [22]. These estimates are far higher than the post-vaccination VP8∗-IgA seropositivity rates seen among vaccinated infants here, strongly suggesting that VP8∗-IgA is not necessary for clinical protection. Similarly, the discordance between total RV-IgG and VP8∗-IgG kinetics suggests that VP8∗-IgG induced by oral vaccination argues against a significant role for VP8∗-IgG in clinical protection following vaccination.

Multiple VP8∗-based parenteral vaccine candidates are currently under evaluation or development [23]. While our results might initially appear to temper enthusiasm for this approach, an important limitation was our inability to test for VP8∗-specific neutralizing antibodies. Since VP4-specific neutralizing antibodies can target either the VP5∗ or VP8∗ subunits [24, 25], the attribution of VP8∗-specific antibodies in human serum or plasma to neutralizing antibody titers using typical neutralization assays is challenging. It is possible (although seems implausible) that natural infection is inefficient at generating high titers of VP8∗-specific neutralizing antibodies relative to total binding antibodies.

Whether very high titers of VP8∗-specific neutralizing antibodies would be sufficient for protection from RV gastroenteritis thus remains unsettled. Such antibodies are highly induced by the parenteral vaccine candidate furthest in clinical development, a trivalent P2-VP8∗ recombinant subunit vaccine developed by PATH [15]. These antibodies are most likely of IgG isotype, which was strongly induced by parenteral vaccination in phase 2 trial, rather than IgA isotype, which was poorly induced. Interestingly, neutralizing antibody titers did not appear to follow a dose-response pattern in this trial, unlike total binding IgG antibodies, although differences in adjusted seroresponse rates were observed for neutralizing antibodies to 1076 (P[6]) strain virus. Vaccinated infants also demonstrated decreased frequency of fecal vaccine shedding when subsequently challenged with Rotarix, suggesting that these antibodies may provide functional mucosal immunity. However, this was based on rates of shedding after the first dose of Rotarix given 4 weeks after completion of study injections, which is later than typical use. In addition, it is well-established that shedding is significantly lower following the second dose of the typical 2-dose Rotarix series [14, 26], so how the degree of mucosal immunity generated by parenteral vaccination compares to that generated by a typical Rotarix schedule remains unclear. Finally, whether neutralizing antibody titer or inhibition of Rotarix shedding correlate with clinical efficacy against RV gastroenteritis also remains to be determined. A phase 3 randomized, controlled trial to address these questions is currently in progress (Clinicaltrials.gov NCT04010448). VP8∗-neutralizing antibodies induced by parenteral VP8∗ immunization would be more likely to act as mechanistic CoPs, as immunity induced by vaccination would be limited to VP8∗-specific responses, compared with the full breadth of anti-RV responses likely to be induced by replicating, live-attenuated ORVs (that more closely simulate natural infection) such as Rotarix.

This study has several additional limitations. Because children in the Natural Infection Study had blood drawn after the onset of diarrhea, it is possible that the VP8∗-binding antibodies detected at day 0 in RV cases were induced by infection and do not represent preexisting antibody concentrations, although lack of correlation between antibodies and DOD at sample collection makes this unlikely. Even if true, such rapid induction would suggest the existence of VP8∗ antigen-specific memory, which would itself argue against the relevance for such responses in clinical protection. Low seropositivity rates for VP8∗-IgA and IgG could indicate suboptimal ELISA sensitivity, but as noted this assay detected VP8∗-IgA at physiologically relevant concentrations during convalescence (day 28) following RV infection. Finally, our findings may not be generalizable to other settings or ORVs.

Correlates of protection for RV, particularly mechanistic correlates, remain elusive. These results suggest that VP8∗-binding antibodies are likely to be non-mechanistic, relative CoPs for RV gastroenteritis but are not necessary for clinical protection following oral Rotarix vaccination and thus are poor candidates for CoPs for future Rotarix vaccine studies. Whether high titers of VP8∗-specific neutralizing antibodies, as may be induced by parenteral VP8∗ vaccines, are sufficient for clinical protection remains unknown and ultimately requires confirmation in prospective clinical trials.

## Supplementary Material

piab120_suppl_Supplementary_Materials

## Notes

***Acknowledgments***. The authors would like to thank Anne Kelsen and Gary Ward for their helpful assistance with protein purification; Benjamin McElvaney, Forida Nazib, Mona Tolba, and Dan Cotnoir for their assistance with assay development and performance; Harry Greenberg for sharing monoclonal antibodies; and Alan Fix and Stan Cryz for sharing VP8∗ parenteral vaccine antigens. The automated DNA sequencing was performed in the Vermont Integrative Genomics Resource DNA Facility and was supported by University of Vermont Cancer Center, Lake Champlain Cancer Research Organization, and the UVM Larner College of Medicine.

***Financial support***. This work was supported by the Bill and Melinda Gates Foundation (grant number OPP1127782 to B. D. K.), the National Institute of General Medical Science at the National Institutes of Health (grant number P20 GM125498 to B. D. K. and B. L.), the Charles H. Hood Foundation Child Health Research Awards Program (to B. L.), the Thrasher Research Fund Early Career Award Program (grant number 13644 to B. L.), and the University of Vermont Internal Grants Program (to B. L).

***Potential conflicts of interest***. All authors: No reported conflicts.
