# Supplementary material for: Plasma VP8∗-Binding Antibodies in Rotavirus Infection and Oral Vaccination in Young Bangladeshi Children
Source: J Pediatric Infect Dis Soc. 2021 Dec 14;11(4):127–33. doi: 10.1093/jpids/piab120 (PMC9055852; doi:10.1093/jpids/piab120)
Supplement: piab120_suppl_Supplementary_Materials [file piab120_suppl_supplementary_materials.docx]

**Supplementary Methods**

P[8] VP8*-specific antibody ELISA

*Antigen generation*

As detailed in **Supplementary Table 1,** the DNA sequence encoding the full-length VP8* segment of Rotarix-strain VP4 was identified (Genbank JN849113.1), codon-optimized for insect cell expression, and synthesized with addition of N-terminus FLAG epitope tag (DYKDDDDK) with a glycine-serine linker and 5’ Not-I and 3’ Xho-I restriction sites (Integrated DNA Technologies, Coralville, IA). The target sequence was cloned into pFastBac1 using the Bac-to-bac baculovirus expression system (Thermo Fisher Scientific, Waltham, MA) per manufacturer’s instructions and confirmed by Sanger sequencing. Generation of recombinant baculovirus for FLAG-VP8* protein expression was performed using the ExpiSf Expression System, according to manufacturer’s instructions (Thermo Fisher Scientific). FLAG-VP8* was purified from infected ExpiSf9 cell lysates by FLAG affinity resin column (Sigma-Aldrich, St. Louis, MO) as previously described [1]. Purified protein was eluted with FLAG peptide or glycine and dialyzed into sterile water. Proper expression was confirmed via detection of an appropriately sized band (28 kDa) by western blot using mouse monoclonal anti-FLAG M2 antibody (Sigma-Aldrich F1804) at 5 ug/mL and mouse monoclonal anti-VP8* clone HS-1 (a kind gift from Harry Greenberg) at 300 ng/mL, incubated overnight at 4^o^C. Secondary detection was performed using HRP-conjugated donkey anti-mouse IgG (ab205724, Abcam, Cambridge, UK) at 1:10,000 dilution for 30 min at room temperature. Blots were developed using SuperSignal West Femto Maximum Sensitivity Substrate (Thermo Scientific 34095), imaged using GE Amersham Iager 600, merged in Fiji 2.1.0/1.53c, and assembled in InkScape 1.0 (**Supplementary Figure 1**). Purified VP8* was lyophilized for long-term storage and reconstituted in sterile water when ready for use. Re-constituted antigen was quantitated using the Pierce BCA Protein Assay kit per manufacturer’s instructions (Thermo Fisher Scientific).

| **Supplementary Table 1.** VP8* antigen sequence | |
| --- | --- |
| Target | Rotarix full-length VP8* (aa 1-246); Genbank JN849113.1 |
| Optimized DNA sequence (5’-3’) | GATCGCGGCCGCATGGACTACAAAGACGATGACGACAAGGGATCCGCTAGCCTCATCTACCGCCAACTCCTGACTAACTCGTACTCAGTGGATCTGCATGACGAAATCGAGCAAATTGGATCCGAGAAGACCCAAAACGTTACCATTAATCCCGGTCCGTTTGCTCAGACGCGCTATGCCCCTGTTAACTGGGACCACGGTGAAATCAATGATTCCACCACAGTGGAGCCTATCCTGGACGGACCATATCAACCCACTACATTCACGCCTCCGAACGACTACTGGATCCTCATTAACTCTAACACAAACGGCGTCGTATACGAGTCGACGAACAACTCAGATTTTTGGACTGCGGTGGTAGCCATCGAGCCTCACGTTAACCCCGTTGACCGCCAGTATATGATATTCGGAGAGAGCAAACAGTTTAACGTGTCTAACGACAGCAATAAGTGGAAGTTCCTGGAAATGTTCCGCTCCTCCTCGCAAAACGAGTTCTATAACCGTCGTACTTTGACTTCTGATACCAGACTCGTGGGTATTTTCAAATACGGAGGTCGCGTCTGGACCTTCCACGGTGAGACTCCCAGAGCTACCACTGACTCAAGTTCTACCGCGAACCTCAACAATATTTCCATAACAATACATTCGGAATTCTACATCATTCCCCGCTCACAGGAAAGCAAGTGCAACGAGTACATTAACAACGGTCTGCCCCCAATCCAGAACACGCGCAACGTTGTGCCATTGCCACTCTCTTCTCGTTCTATCCAATACAAGCGTTAGCTCGAGGATC |
| Protein translation (5’-3’)  **FLAG** linker | M**DYKDDDDK**GSASLIYRQLLTNSYSVDLHDEIEQIGSEKTQNVTINPGPFAQTRYAPVNWDHGEINDSTTVEPILDGPYQPTTFTPPNDYWILINSNTNGVVYESTNNSDFWTAVVAIEPHVNPVDRQYMIFGESKQFNVSNDSNKWKFLEMFRSSSQNEFYNRRTLTSDTRLVGIFKYGGRVWTFHGETPRATTDSSSTANLNNISITIHSEFYIIPRSQESKCNEYINNGLPPIQNTRNVVPLPLSSRSIQYKR |

**
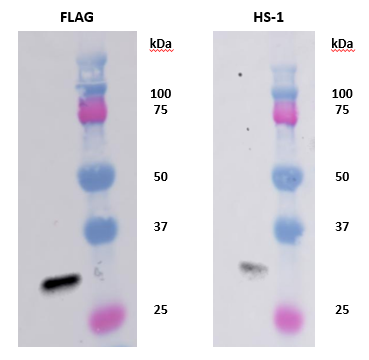
**

**Supplementary Figure 1.** Western blots of purified FLAG-VP8* detected using anti-FLAG M2 antibody (left) and anti-VP8* clone HS-1 (right).

*VP8*-IgA and VP8*-IgG ELISA procedure and validation*

96-well microtiter ELISA plates (Corning Costar 9018, Corning, NY) were coated with 30 ng/well of purified antigen (odd columns) or matching control (even columns) in carbonate-bicarbonate coating buffer (pH 9.4-9.8). Control wells consisted of an equivalent concentration (w/w) of FLAG peptide and mock-infected Sf9 cell lysate to correct for potential off-target binding to either FLAG epitope tag or residual Sf9 insect cell protein contamination. After overnight incubation at 4°C, plates were washed 5 times in wash buffer containing 0.05% Tween then blocked in 1% (w/v) skim milk (Oxoid Ltd., Hampshire, England) in wash buffer for 60 minutes at 37°C on an orbital shaker at 140 rpm. Following blocking, plates were washed and antigen and control wells were incubated with 50 uL/well of each plasma specimen at the appropriate dilution in 1% skim milk for 60 minutes at 37°C at 140 rpm. Plates were washed and incubated for 30 minutes at 37°C at 140 rpm with biotinylated rabbit anti-human IgA (Jackson ImmunoResearch, West Grove, PA, #135073) diluted 1:1000 in 1% skim milk or anti-human IgG (Jackson ImmunoResearch #135073) diluted 1:2000. Plates were washed and incubated with avidin-biotin peroxidase complex (Vectastain Elite ABC-HRP standard kit, Vector Laboratories, Inc, Burlingame, CA) diluted 1:1000 in wash buffer for 30 minutes at room temperature at 140 rpm. Plates were washed 5 times in wash buffer and once in citric acid phosphate buffer (pH 4.8-5.2) followed by addition of O-phenylenediamine dihydrochloride (OPD) substrate prepared according to manufacturer’s instructions (Sigma-Aldrich, SLBW6325) for 60 minutes at room temperature, protected from light. The reaction was then stopped with 0.1M sulphuric acid and optical density (OD) measured at 490 nm. Corrected OD was calculated by subtracting the background OD value from the control well from the OD value in the corresponding antigen-containing well for each specimen dilution.

Specimens obtained from healthy adult volunteers were screened to identify suitable samples for use as reference standards to generate eight-point standard curves using two-fold (IgA) or three-fold (IgG) serial dilutions beginning at 1:20. Additional specimens were selected for use as positive and negative controls. The standards were assigned a value of 1000 arbitrary units (U), and antibody concentrations were interpolated from corrected OD values using a 4-parameter logistic (4PL) regression using Gen5 software, version 3.03 (BioTek Instruments, Inc., Winooski, VT). Different specimens were identified and used as standards and controls for the IgA and IgG assays, thus the interpolated concentrations for each assay are not directly comparable. The lower limit of detection (LLOD) for IgA was determined to be 10 U/mL and for IgG was 11 U/mL, calculated as the mean plus 3 standard deviations of multiple replicates of a negative control plasma specimen.

Following validation, each plasma specimen was assayed initially at dilutions of 1:20, 1:40, 1:80, and 1:160, or higher as necessary for IgA and 1:50, 1:100, 1:200, and 1:400, or higher as necessary for IgG. Results were accepted if at least two dilutions gave consistent measurements with a coefficient of variance of <25% and the standard curve and controls on each plate met pre-specified parameters. All negative specimens were assigned a value at the LLOD.

P[4] and P[6] VP8*-IgA ELISA

96-well microtiter plates were coated with 30 ng of P[4] P2-VP8* or P[6] P2-VP8* in carbonate-bicarbonate buffer and incubated overnight at 4°C. Plates were washed 5 times in wash buffer containing 0.05% Tween then blocked in 1% (w/v) skim milk (Oxoid) in wash buffer for 60 minutes at 37°C on an orbital shaker at 140 rpm. Following blocking, plates were washed and two-fold serial dilutions in 1% skim milk of each plasma specimen starting at 1:5 were added for 60 minutes at 37°C at 140 rpm. All subsequent steps proceeded as described above for P[8] VP8*-IgA ELISA. End-point titer was defined as the highest dilution giving an OD value greater than the mean plus 3 standard deviations of multiple replicates of the corresponding dilution of negative control.

RV-IgG ELISA

An ELISA to measure total RV-specific IgG in human plasma was adapted from a previously described assay to measure RV-IgA with minor modifications [2]. 96-well microtiter plates were coated with polyclonal rabbit anti-rotavirus IgG (Dako North America, Inc O9187, Carpinteria, CA) in carbonate-bicarbonate coating buffer (pH 9.4-9.8) and incubated overnight at 4⁰C. Plates were washed five times in wash buffer containing 0.05% Tween and then incubated with clarified supernatants of strain 89-12 rotavirus virus lysate propagated in MA104 cells *(*odd columns) or mock-infected cell lysates (even columns) diluted in 1% (w/v) skim milk at 37⁰C for 2 hours on an orbital shaker at 140 rpm. Plates were washed then serial dilutions in 1% skim milk of each specimen were added to matching viral lysate/control wells and incubated at 37⁰C for 60 minutes on an orbital shaker at 140 rpm. Plates were washed then incubated with biotinylated rabbit anti-human IgG (Jackson Immunoresearch #135073) at a dilution of 1:1000 in 1% skim milk at 37⁰C for 30 minutes on an orbital shaker at 140 rpm. Plates were again washed and incubated with avidin-peroxidase complex (Manufacturer) diluted 1:4000 in wash buffer for 30 minutes at room temperature at 140 rpm. Plates were then washed five times with wash buffer and once in citric acid phosphate buffer (pH 4.8-5.2) followed by addition of OPD substrate for 30 minutes at room temperature, protected from light. The reaction was then stopped with 0.1M sulphuric acid and optical density (OD) measured at 490 nm. Corrected OD was calculated by subtracting the background signal from the mock-infected lysate (control) well from the OD value in the corresponding viral lysate well for each specimen dilution.

The ELISA underwent validation as described above for VP8* ELISA. A different reference specimen was selected for standard curve generation than was used for RV-IgA, and was assigned a value of 1000 arbitrary units. Thus, RV-IgA and RV-IgG concentration cannot be directly compared. The lower limit of detection for RV-IgG was determined to be 9.5 U/mL. Specimens were initially assayed at dilutions of 1:1000, 1:2000, 1:4000, and 1:8000, or higher as necessary. Results were accepted if at least two dilutions gave consistent measurements with a coefficient of variance of <25%.

**References**

1. Bookwalter CS, Kelsen A, Leung JM, Ward GE, Trybus KM. A Toxoplasma gondii class XIV myosin, expressed in Sf9 cells with a parasite co-chaperone, requires two light chains for fast motility. J Biol Chem **2014**; 289(44): 30832-41.

2. Lee B, Dickson DM, Alam M, et al. The effect of increased inoculum on oral rotavirus vaccine take among infants in Dhaka, Bangladesh: A double-blind, parallel group, randomized, controlled trial. Vaccine **2020**; 38(1): 90-9.
